# Environmental Factors Associated with Mosquito Vector Larvae in a Malaria-Endemic Area in Ratchaburi Province, Thailand

**DOI:** 10.1155/2018/4519094

**Published:** 2018-12-18

**Authors:** Tanawat Chaiphongpachara, Prasit Yusuk, Sedthapong Laojun, Chaekki Kunphichayadecha

**Affiliations:** ^1^College of Allied Health Science, Suan Sunandha Rajabhat University, Thailand; ^2^Bachelor of Public Health, College of Allied Health Sciences, Suan Sunandha Rajabhat University, Thailand

## Abstract

Malaria is transmitted by female mosquitoes in the genus* Anopheles* and is a major public health issue. Different species of* Anopheles* mosquitoes have different epidemiological characteristics, behaviors, and ecological requirements, and so an understanding of their biology and ecology in a particular area is critical for successful disease control. The aim of this study was to determine which environmental factors are associated with* Anopheles* larvae in a malaria-endemic area in Ratchaburi Province, Thailand, which shares a border with Myanmar. In October 2016, we collected mosquito larvae and measured six environmental factors at 10 study sites located along Lam Pachi River, which flows through Huay Nam Nak village in Ratchaburi Province. We found two species of* Anopheles* larvae (*An. subpictus* sensu lato (s.l.) Grassi and* An. barbirostris* s.l. van der Wulp) at 7 of the 10 study sites, the numbers of which significantly differed between sites (*p* < 0.05). Pearson correlation analysis showed that the numbers of larvae of both species were significantly positively correlated with the dissolved oxygen level (*p *< 0.01) and significantly negatively correlated with the width of the river (*p* < 0.05) and pH (*p *< 0.01). By contrast, turbidity, water depth, and water temperature were not associated with larval abundance. Mosquito species which belong to genus* Anopheles* are considered to be of public health and medical importance. Therefore,* Anopheles* mosquito surveillance and control in the study sites are essential. This information will facilitate vector-borne disease control and improve our understanding of the biology of* Anopheles *vectors in rivers located along international borders, further reducing the number of patients in this malaria-endemic area.

## 1. Introduction

Malaria is a mosquito-borne disease that is caused by protozoan parasites of the genus* Plasmodium*, five species of which are responsible for human malaria:* P. falciparum*,* P. vivax*,* P. ovale*,* P. malariae*, and* P. knowlesi *[1]. This disease is a major public health issue, with approximately 212 million cases and 429,000 associated deaths occurring worldwide in 2015 [2]. Malaria is a particular problem in tropical and subtropical areas [3], including Thailand, where there were 11,528 cases across 73 provinces in 2014, with a morbidity rate of 17.88 per 100,000 population and three deaths as a result of this disease (Bureau of Epidemiology of Department of Disease Control) [4].

Malaria is transmitted by female mosquitoes of the genus* Anopheles*. This genus includes approximately 484 species, but only around 100 of them can transmit* Plasmodium* to humans [5]. Approximately 74 species of* Anopheles* vectors have been recorded in Thailand [6], which are divided into three groups: primary vectors, which include* An. dirus* Peyton & Harrison,* An. minimus *Theobald, and* An. maculatus* Theobald; secondary vectors, which include* An. epiroticus *Linton & Harbach,* An. aconitus *Doenitz, and* An. pseudowillmori* Theobald; and suspected vectors, which include* An. philippinensis* Ludlow,* An. barbirostris* van der Wulp,* An. campestris *Reid, and* An. culicifacies *Giles. Different species of* Anopheles* have different epidemiological characteristics, behaviors, and susceptibility to* Plasmodium *species and insecticides [7]. Therefore, an understanding of the biology and ecology of malaria vectors in the area of interest is critical for successful disease control [8–10].

The larval stages of all species of* Anopheles *require water, so the presence of a water source is associated with an increased distribution and density of larvae, as well as an increased incidence of adult mosquitoes and thus malaria [11, 12]. Several factors are correlated with the presence of larvae, including pH, temperature, dissolved oxygen (DO) level, alkalinity, and phosphate and chloride concentrations [12]. Moreover, different species occupy different habitats, with* An. minimus *and* An. maculatus *preferring small or moderate-sized streams with slow running, clear water as breeding sites, and* An. dirus* preferring small, shallow, rain-filled, transient, shady puddles, and ground pools [13]. In Thailand, the primary vectors live in forests, so malaria-endemic areas tend to be associated with forest habitats [14].

The highest epidemic area in Thailand occurs along international borders with Myanmar, accounting for approximately 85% of all reported cases in the country [15]. Ratchaburi Province is located on the western border of Thailand adjacent to Myanmar and has a high incidence of malaria, with a morbidity rate of 8.95 per 100,000 population in 2016 (Bureau of Epidemiology) [16]. The Lam Pachi River is an important river that flows through many villages in Ratchaburi, where it divides into several small streams, providing good habitat for* Anopheles *mosquitoes. However, little is currently known about the larval biology of* Anopheles *species in this river.

The aim of this research was to determine which environmental factors are associated with* Anopheles* larvae in the Lam Pachi River. The results of this research will provide useful information about the distribution patterns of* Anopheles *mosquito larvae, allowing mosquitoes to be controlled around rivers that cross international borders and leading to improved vector-borne disease control.

## 2. Materials and Methods

### 2.1. Study Sites

**Figure 1:**
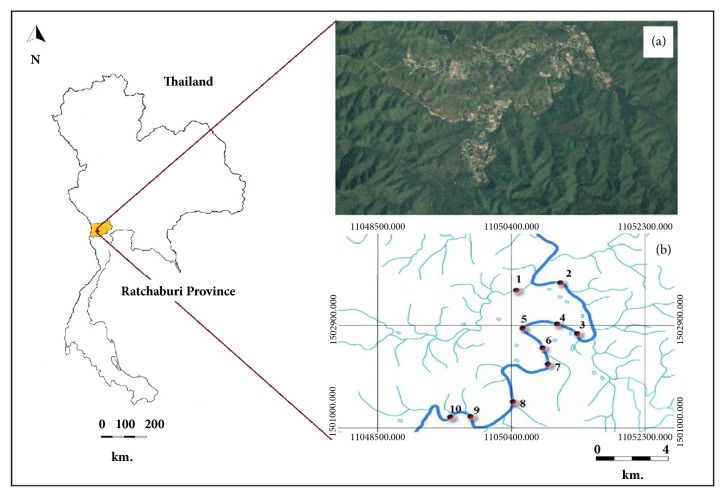
Location of the study area and sites. (a) Huay Nam Nak village, Thailand, and (b) the 10 study sites in the Lam Pachi River that were used for the larval survey.

This study was conducted in the part of the Lam Pachi River that flows through Huay Nam Nak village (13°22′36.0^″^N 99°16′34.9^″^E) in the Suan Phueng District of Ratchaburi Province, Thailand (see Figure 1(a)). In 2016, the Bureau of Infectious Diseases reported that this was an epidemic area for malaria, with a morbidity rate of 8.95 per 100,000 population. This village is composed of wooden houses and agricultural fields, is surrounded by mountains and hilly forests, and has the Lam Pachi River flowing through it.

We collected mosquito larvae and measured environmental factors at 10 sites along the Lam Pachi River (Table 1 and Figure 1(b)). These sites were selected based on the criteria that they were approximately 1–4 km apart and could be explored (some areas were in deep forest and valleys).

### 2.2. Larval Mosquito Collection, Rearing, and Species Identification

Mosquito larvae were collected from each of the 10 study sites in October 2016. A total of 20 dips were taken from the available river at each study site between 8:00 and 11:00 AM using a standard mosquito dipper and were collected into separate bottles for each location. Three replicate samples were taken from each study site. The number of larvae was then counted, and the bottles were labeled with the collection date and site and transported to the laboratory of the College of Allied Health Science, Suan Sunandha Rajabhat University, Samut Songkhram Center, Thailand. The collected larvae were reared in plastic trays (separated by location) containing breeding water and were provided with rabbit food each day. The trays were maintained at approximately 25°C and 55% relative humidity. Once the pupal stage had been reached, the mosquitoes were transferred to 30 × 30 × 30-cm cages (separated by location) to facilitate adult emergence. Following emergence, the adult mosquitoes were killed by placing them in a freezer at −20°C for 20 min, and the male mosquitoes were separated from the female mosquitoes. The mosquitoes were then identified using “Illustrated keys to the mosquitoes of Thailand” [17] and counted.

### 2.3. Creation of Anopheles Larva Surveillance Map in the Areas along the Phachi River Flowing through Huay Nam Nak Village

In this study, to create a map of* Anopheles* larva surveillance, we used Qgis, free map design software which can be downloaded at https://www.qgis.org/en/site/. We put two sets of data into the map including the information regarding land use, kindly supported by Land Development Department of Thailand, as well as the information regarding* Anopheles *larva distribution, used as a percentage, from our survey.

### 2.4. Measurement of Environmental Factors in the River

Six environmental factors were measured in triplicate at each of the 10 study sites where mosquito larvae were collected, approximately 30 cm away from the riverbank: turbidity, water depth, river width, water temperature (WT), pH, and DO level. The turbidity of the water was measured with a turbidity meter (HI93703C; Hanna Instruments, USA), the river depth and width were measured with a measuring rod (100 cm) and measuring tape (1000 cm), and the water quality parameters WT, pH, and DO were measured with a water quality meter (WA-2017SD Multi parameter Display System; Lutron, Taiwan). All measurements were made immediately after larval collection.

### 2.5. Statistical Analyses

Means and standard deviations were calculated for the number of* Anopheles *mosquitoes collected for each species and for each environmental factor that was measured. Differences in the numbers of* Anopheles* mosquitoes of each species that were collected from each site were analyzed using one-way analysis of variance (ANOVA) followed by post hoc tests. Furthermore, Pearson correlation analysis was used to examine the relationship between each of the environmental factors in the breeding habitat and the abundance of mosquito larvae from each species. All data analyses were conducted using SPSS version 17 (SPSS Inc., Chicago) with a significance level of* p* < 0.05.

## 3. Results

### 3.1. Distribution of Mosquito Larvae

We found two species of* Anopheles *larvae at 7 of the 10 study sites:* An. subpictus* sensu lato (s.l.) Grassi and* An. barbirostris* s.l. van der Wulp (Table 2). No* Anopheles *larvae were found at the remaining three locations (sites 5, 8, and 10). At all seven sites,* An. subpictus* s.l. was present at a higher abundance than* An. barbirostris* s.l. However, there was also a significant difference in the number of mosquito larvae among sites (ANOVA,* p* < 0.05; Table 2).

### 3.2. Map of Anopheles Larva Surveillance in the Areas along the Phachi River Flowing through Huay Nam Nak Village

**Figure 2:**
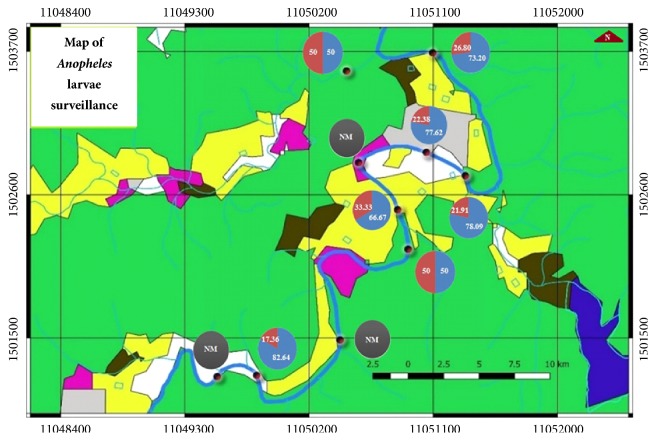
Map of* Anopheles* larva surveillance in the areas along the Phachi River flowing through Huay Nam Nak village (in a circle, red = percentage of* An. barbirostris* s.l., blue = percentage of* An. subpictus* s.l., and black = no mosquito larvae (NM)) (physical environment characteristics: green = forest area, yellow = agricultural land, black = thick forest, pink = orchard, blue = reservoir, and gray = residential area).

The map of* Anopheles* larva surveillance in the areas along the Phachi River flowing through Huay Nam Nak village clearly showed patterns of* Anopheles* larva distribution (Figure 2).

### 3.3. Variation in Environmental Factors among Sites

**Figure 3:**
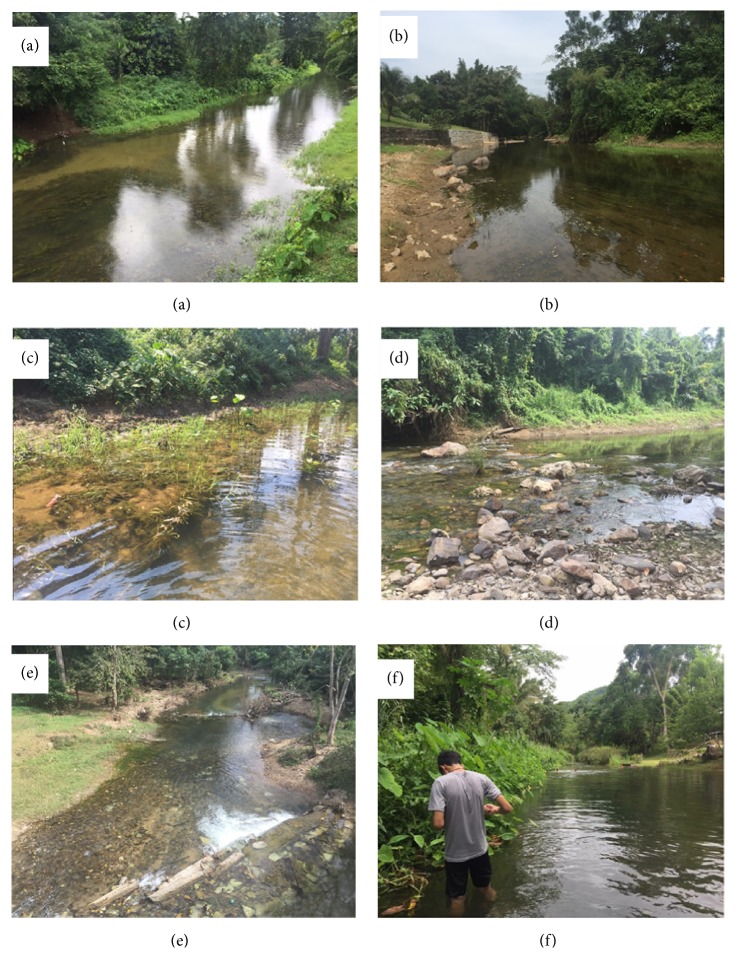
Characteristics of the study sites in the Lam Pachi River, Thailand. Some locations had (a) a high water depth, including sites 5, 8, and 10; (b) a calm stream, including sites 2 and 3; (c) many plants along the riverbank; (d) many rocks, including site 10; (e) a low water depth, including sites 1 and 9; and (f) high water turbidity, including sites 7 and 8.

We found that turbidity was highest at site 9 and lowest at site 1, water depth was highest at site 5 and lowest at site 1, river width was highest at site 7 and lowest at site 1, and DO was highest at site 3 and very low at sites 1, 7, and 8 (Table 3). Neither the WT nor pH varied greatly among sites (WT, range = 29.13°C–30.97°C; pH, range = 7.23–8.23). Some of the variation in river characteristics between sites is shown in Figure 3.

### 3.4. Relationship between Environmental Factors and Mosquito Larval Abundance

The larval abundances of both* An. subpictus* s.l. and* An. barbirostris *s.l. were significantly positively correlated with DO (*p *< 0.01) and significantly negatively correlated with pH (*p *< 0.01) and river width (*p* < 0.05; Table 4). By contrast, larval abundance was not associated with turbidity, water depth, or WT for either species.

## 4. Discussion

**Figure 4:**
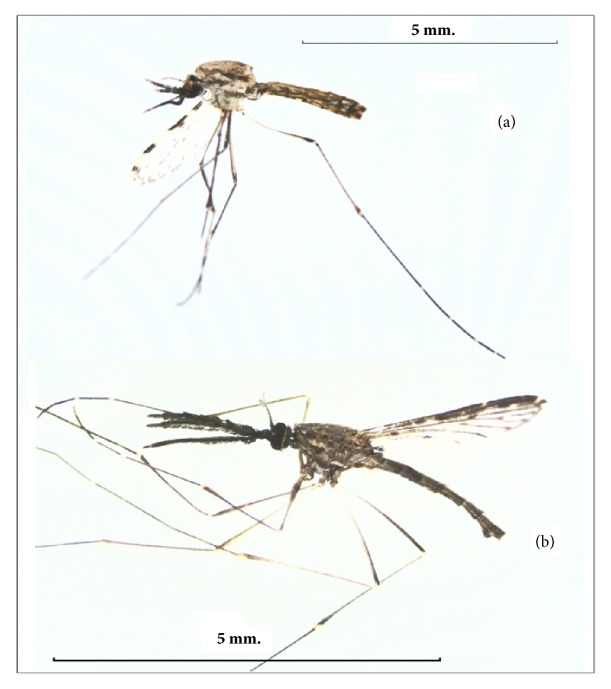
Adults of the two* Anopheles* species found in the study area. (a)* An. subpictus* s.l. Grassi, which is a malaria vector in Sri Lanka, and (b)* An. barbirostris* s.l. van der Wulp, which is a suspected malaria vector in Thailand.

In this study, we collected water samples from 10 sites in the Lam Pachi River, which runs through the malaria-endemic area of Ratchaburi Province, Thailand, in October 2016 and found two species of* Anopheles *larvae:* An. subpictus* s.l. and* An. barbirostris *s.l. In addition, we found culicine mosquitoes such as* Cx. quinquefasciatus* Say,* Cx. whitmorei* Giles, and* Cx. vishnui* Theobald at some surveyed areas. However, we did not study them because the* Culex* mosquitoes are not the vectors of malaria.* Anopheles subpictus *s.l. is not a proven vector of malaria in Thailand but is a known vector in Sri Lanka [18]. By contrast,* An. barbirostris* s.l. is a suspected vector of malaria and filariasis and has been reported to have transmitted* Plasmodium vivax *infection to humans in Tak Province [9] (Figure 4). Moreover,* An. barbirostris* s.l. is a vector of* P. falciparum* in Bangladesh and Sri Lanka [19, 20]. Both species are recognized as being species complexes. However, their classification currently requires molecular techniques due to their highly similar morphologies [21, 22] unapplied in this study, so we were unable to identify these in this study.

Our results are consistent with previous findings that* An. subpictus *s.l. and* An. barbirostris* s.l. inhabit a variety of aquatic habitats in India and Peninsular Malaysia, including ponds, pools, rice fields, rivulets, straw fields, borrow pits, ditches, irrigation channels, wells, and seepages [23, 24]. Furthermore, the presence of larvae of both species has been reported in rivers in Sri Lanka, most of which were also* An. subpictus* s.l. rather than* An. barbirostris* s.l. [24]. We did not find the larvae of any primary malaria vectors, including* An. dirus*,* An. minimus*, and* An. maculatus*, in the study area, probably because this water source was unsuitable habitat for them; most of the habitat in this area is slow-flowing streams with clear water, often in forests or near hilly and mountainous areas [25].

The physical and chemical properties of* Anopheles *breeding sites have both direct and indirect effects on their biology, including oviposition, survival, and spatial distribution [26]. In this study, we found that the abundance of mosquito larvae was significantly correlated with pH, DO, and river width across the study sites.

pH was significantly negatively associated with the abundance of both* An. subpictus *s.l. and* An. barbirostris* s.l. larvae, supporting previous reports that* Anopheles* larvae prefer a neutral pH (approximately pH 7.0) [27] and that very low pH values in the laboratory have a severe effect on larvae, including 100% mortality within 24 h at pH 3.0 and developmental delays at pH 4.0 [28].

DO was significantly positively associated with the abundance of both species, indicating that the larvae prefer high DO levels. DO content is an index of water quality, with higher values corresponding with cleaner water [29]. Most species of* Anopheles* prefer clean water in the wild, which contrasts with* Culex *mosquitoes, which can adapt to polluted water [30].

Finally, the width of the river was significantly negatively correlated with the abundance of both species. Larger rivers tend to have stronger currents, which are very dangerous to mosquito larvae [12]. Consequently, mosquito larvae tend to be associated with stagnant and slow running water in the wild, and nearly all larvae will be eliminated by currents of >15 kilogallons/min [12, 27].

Interestingly, turbidity, WT, and water depth were not associated with the number of mosquito larvae in the study area, despite all three factors having previously been shown to be related to* Anopheles *larvae. Previous research has found that the turbidity of the water is related to larval abundance [31], WT affects the life history of* Anopheles *mosquitoes during the larval and adult stages [32], and water depth is related to the presence of aquatic plants, which provide larval habitat and shelter. However, it has also been shown that the effects of these factors vary depending on the habitat specificity of the species being examined [33].

To succeed in making effective plans for vector mosquito control, it highly depends on understanding of mosquito habitats. Our created map of* Anophel*es larva surveillance in the areas along the Phachi River flowing through Huay Nam Nak village (Figure 2) was designed to be easy to understand in order to be implemented in making effective plans for vector mosquito control and* Anopheles *larva habitat destruction. The map depicts the overall areas in a large scale to show the environment that mosquitoes live, especially in the forest.

## 5. Conclusions

We found that the larvae of* An. subpictus* s.l. and* An. barbirostris *s.l. occurred at 7 of the 10 sites that were investigated along the Lam Pachi River where it flows through Huay Nam Nak village and that pH, DO level, and river width were significantly associated with larval abundance. This information adds details on the biology of* Anopheles* species present in our study area and will help to control malaria, a disease here endemic. Although the larvae of* Anopheles* species including* An. dirus* Peyton &amp; Harrison,* An. minimus* Theobald, and* An. maculatus* which are proven as primary vectors of malaria in Thailand were not found, mosquito species belong to genus* Anopheles *are considered to be of public health and medical importance, especially for transmitting malaria and a few species of their association with filariasis and arbovirus infections. Potential vectors of malaria are different in each geographical range. Some species of* Anopheles *mosquitoes are considered as primary vectors in some areas but are secondary vectors in other areas.* An. barbirostris* is a suspected vector and a potential vector of malaria in Thailand. Reducing the number of* Anopheles* population in nature is important. In addition,* Anopheles* mosquito surveillance and control are essential. There are several ways to control larvae of* Anopheles* such as disposing weeds at the edge of water which is a primary habitat.

## Figures and Tables

**Table 1:** Coordinates of the 10 sites used for the larval survey.

Site	Latitude	Longitude
1	13°22′57.58^″^N	99°16′9.33^″^E
2	13°23′4.42^″^N	99°16′25.24^″^E
3	13°22′35.55^″^N	99°16′31.70^″^E
4	13°22′40.47^″^N	99°16′23.05^″^E
5	13°22′26.92^″^N	99°16′16.68^″^E
6	13°22′16.55^″^N	99°16′18.93^″^E
7	13°21′52.26^″^N	99°16′3.48^″^E
8	13°21′46.94^″^N	99°15′58.91^″^E
9	13°21′47.29^″^N	99°15′43.16^″^E
10	13°21′44.95^″^N	99°15′33.83^″^E

**Table 2:** Number of *Anopheles *larvae at the 10 study sites in the Lam Pachi River, Thailand.

Site	Means±SD of *Anopheles *larvae		
	*An. subpictus* s.l.	*An. barbirostris* s.l.	Both species
1	0.33±0.58^a^	0.33±0.58^a^	0.66±0.58^a^
2	33.67±2.52^b^	12.33±2.52^b^	46.00±5.00^b^
3	19.00±3.61^c^	5.33±4.13^c^	24.33±7.51^c^
4	19.67±3.51^c^	5.67±3.21^c^	25.33±1.57^c^
5	0.00±0.00^a^	0.00±0.00^a^	0.00±0.00^a^
6	0.66±0.58^a^	0.33±0.58^a^	1.00±0.00^a^
7	0.33±0.58^a^	0.33±0.58^a^	0.67±0.58^a^
8	0.00±0.00^a^	0.00±0.00^a^	0.00±0.00^a^
9	6.33±2.08^d^	1.33±0.58^d^	7.67±2.31^d^
10	0.00±0.00^a^	0.00±0.00^a^	0.00±0.00^a^

Total	8.00±11.63	2.57±4.22	10.57±15.60

SD, standard deviation. Values within the same column with different superscript letters are significantly different (ANOVA, *p* < 0.05). All values are means ± SD.

**Table 3:** Environmental characteristics of the 10 study sites in the Lam Pachi River, Thailand.

Site	Means±SD of environmental factor					
	Turbidity (FTU)	water depth (cm)	width of river (cm)	WT (°C)	pH	DO (mg/L)
1	26.66±2.08	17.66±3.51	109.67±11.50	29.13±1.10	8.13±0.06	1.43±0.06
2	27.33±1.53	31.00±6.56	473.00±42.19	30.13±0.95	7.23±0.06	6.23±0.06
3	28.33±0.58	33.00±2.65	486.67±37.90	30.73±0.81	7.27±0.12	6.37±0.06
4	29.33±1.53	28.33±4.93	582.33±57.93	29.87±0.35	7.23±0.15	6.40±0.10
5	30.00±1.00	57.33±2.52	454.00±37.00	30.57±0.47	8.17±0.06	1.47±0.06
6	28.33±1.53	46.33±3.79	374.33±66.61	30.55±0.70	7.23±0.15	6.13±0.06
7	38.00±2.65	47.33±3.51	606.33±76.64	30.37±0.86	8.23±0.15	1.43±0.06
8	39.33±2.52	56.67±3.79	457.33±58.53	30.97±0.32	8.13±0.06	1.43±0.06
9	28.00±1.00	23.67±3.78	509.33±31.09	29.47±0.60	7.73±0.06	4.07±0.15
10	29.67±1.15	57.67±4.51	423.66±36.67	30.10±0.89	8.23±0.15	1.47±0.06

SD, standard deviation; WT, water temperature; DO, dissolved oxygen.

**Table 4:** Relationship between *Anopheles *larval abundance and environmental factors in the Lam Pachi River, Thailand.

*Anopheles larvae*	Means±SD of each environmental factor in river					
	Turbidity (FTU)	Water depth (cm)	Width of river (cm)	WT (°C)	pH	DO (mg/L)
***An. subpictus *s.l.**						
* r*	-0.334	0.284	-0.444^*∗*^	-.012	-0.741^*∗∗*^	0.741^*∗∗*^
* p*	0.072	0.129	0.014	.950	< 0.001	< 0.001
***An. barbirostris *s.l..**						
* r*	-0.282	0.264	-0.407^*∗*^	.020	-0.640^*∗∗*^	0.652^*∗∗*^
* p*	0.131	0.159	0.026	.917	< 0.001	< 0.001
**All species**						
* r*	-0.325	0.283	-0.441^*∗*^	-.004	-0.725^*∗∗*^	0.736^*∗∗*^
* p*	0.080	0.130	0.015	.985	< 0.001	< 0.001

FTU, formazin turbidity unit; WT, water temperature; DO, dissolved oxygen. ^*∗*^*p* < 0.05; ^*∗∗*^*p* < 0.01 (two-tailed Pearson correlation).

## Data Availability

The data used to support the findings of this study are available from the corresponding author upon request.
